# Scotland's Record Marriage Figures

**Published:** 1920-11-27

**Authors:** 


### Scotland's Record Marriage Figures.

Will an increase in the marriage-rate help to compensate for the growing practice of birth control, and so help to keep the birth-rate high? The question is suggested by the figures given in the annual report of the Registrar-General for Scotland, now issued for the year 1919. In that year the number of marriages registered and the marriage-rate were the highest on record. The number, 44,137, is 9,608 more than in the preceding year, 10,611 more than the mean of the figures for five preceding years, and 11,470 more than the mean of those for ten preceding years. The frequency of irregular marriage in Scotland continues high. Some of the most striking figures in the report are the following:—Two brides of 15; 5 bridegrooms of 16; 21 bridegrooms under 20 married widows; 4 brides (widows) aged over 70; five bridegrooms of 80 and over. There were 111 bridegrooms aged 17; 369 aged 18; and 621 aged 19. What would Mrs. Marie Stopes have to say about such figures as these?

The population of Scotland in 1919 is estimated at 4,894,077—133,173 more than in 1911; males, 2,373,422; females, 2,520,655. The births numbered 106,268, or 7,714 more than in 1918; and the deaths 75,149, or 3,223 less than in the preceding year.

